# The National Clean Milk Society's Investigation

**Published:** 1917-06-09

**Authors:** 


### MILK FOR INFANTS AT SCHOOLS FOR MOTHERS.

### The National Clean Milk Society's Investigation.

The drastic reforms in the conditions of our milk supply which were impending in 1914 had, like much else, with the beginning of the war to be put aside. Milk has since been frequently a subject of discussion, but the question has been far more often one of supply or of price than of purity; and, whilst the cost of milk to the consumer has had to be increased, there has been no commensurate improvement in its quality or cleanliness. In fact, with inevitable relaxation of supervision, the state of things is undoubtedly to-day rather worse than better.

The National Clean Milk Society, to the good work of which we have often called attention, has recently made an investigation into the hygienic quality of the milk purchased for the babies attending at certain "schools for mothers." Inquiry was also made concerning the milk supplied at such schools, at which (if anywhere) we are justified in hoping to find exercised an exceptional care in selection. The results of the Society's inquiries are somewhat disheartening; they "afford further proof, if any be needed, that for all practical purposes the subject of clean, hygienic milk has been ignored in England. The lack of action on the part of welfare workers is probably due to the immense problem that confronts them," a problem the solution of which is properly the business of the Public Health authorities. We are not surprised by the result of the investigation, and we fully sympathise with the welfare workers in our great towns, who have no facilities for obtaining, except in very rare instances, and then only at a cost almost prohibitive, a milk supply even fairly reliable, still less of maintained excellence. The Society, it is true, makes some suggestions, but they are necessarily brief and incomplete. We fear that the welfare worker seeking to act upon them, in face of milk-trade interests and complexities, is likely to meet with disappointment and to realise her limitations.

### Milk Control in America.

The Society is conversant with the methods in vogue in America for the control and improvement of the milk supply which were the subject of a report to the Local Government Board by Dr. Arthur Eastwood in 1909. Dr. Kerr, of the United States Public Health Service, classified the supervision of milk supplied in the United States in point of time in three epochs—namely, from 1855 to 1880, during which time the object was essentially the prevention of fraud; from 1880 to 1890, when attention was devoted primarily to dangerous milk; from 1890 to 1913, during which time the object has been mainly the production of safer milk. We, in England, have not yet got beyond the first stage, and are, therefore, some thirty years behind America. We have done but little beyond attempting to secure the public against the theft of cream from the milk; leaving it to be supposed that the worst, if not the only, offence which the dairyman can commit is to water the "pure" milk or to substitute "skimmed" milk for it: the dairyman has learnt the use of the separator, and "tones" milk as a routine process. Infected, dirty, stale, and "warm" milk is freely sold for use by the public, including the babies. It is, with a frequency infinitely too great, infected with tubercle as the result of the acknowledged presence in milking-herds of tuberculous cows; whilst bacilli of the coli group are almost invariably present in it. Milk is now carried great distances by rail, and is consequently stale before it reaches the consumer, no means having been meanwhile adopted to keep it cool. The various organisms it inevitably contained at the farm become multiplied, in so excellent a medium, in a degree beyond imagination. The bacterial content of an average sample of London milk is greatly above the American standard; in fact it would be illegal to sell such milk in New York, Philadelphia, or many other American cities.

### The Milk in Schools for Mothers.

In answer to questions addressed to the managers of schools for mothers concerning the milk there supplied, the Society obtained information from eighty-three such centres. Sixty-one of these supplied milk: this was dried milk in thirty-six cases, raw milk in thirteen, condensed in two, boiled in four, pasteurised in two, and separated in one. Raw cow's milk is, without doubt, the best basis for the food of infants not fed at the breast, if only it can be obtained fresh and clean from healthy cows. If the schools which use it have facilities for obtaining a really reliable supply, they and their infants are exceptionally fortunate. Four schools boil and two "pasteurise" their milk, no doubt hoping by so doing to lessen the risks of tuberculous infection and diarrhœal diseases from its use. Both these methods damage the milk as a food for infants, and their adoption is a confession of weakness. Milk boiled to sterilisation is, for babies, milk all but spoiled. Pasteurisation is a difficult process, uncertain in its results—especially when attempted under domestic conditions: it is by no means unknown to the milk trade; pasteurised stale milk is frequently mixed with some comparatively fresh, and the mixture sold as "new" milk.

### Dried Milk.

It is of interest that no fewer than thirty-six of these schools for mothers supply dried milk. The demand for dried milks has grown very rapidly of recent years, and is still growing, although they are relatively expensive, which fact shows that the public will pay a higher price for milk in the quality of which it has confidence, which is obtainable in a convenient form, and in the use of which there is no waste. Of the dried milks now on the market, both full and separated, many are excellent preparations. The dangers of handling and delay incidental to the distribution of milk in the usual way are in great measure avoided in the case of these desiccated milks. The methods by which they are prepared include an effective pasteurisation, and have to be carried out with care and accuracy in order that a marketable standard of the commodity may be maintained. There is competition in the sale of the different brands, and the consumer can, in some degree, form a judgment of their relative values. The dried milks of good quality are finely powdered, are almost white in colour, and are cleanly packed in airtight tins. They have obvious advantages in use, especially those of comparative safety and regularity of hygienic quality. Few unbiassed observations of the results obtained from their continued use in infant feeding have been published; but we believe that they are proving, and will continue to prove, satisfactory. In comparison, "new milk" as ordinarily sold in towns is frequently unsuitable and even dangerous.

